# GLP-1RAs in patients with psoriasis

**DOI:** 10.1007/s42000-025-00635-5

**Published:** 2025-02-20

**Authors:** Ioanna A. Paschou, Evangelia Sali, Stavroula A. Paschou, Theodora Psaltopoulou, Electra Nicolaidou, Alexander J. Stratigos

**Affiliations:** 1 1st Department of Dermatology and Venereology, “Andreas Sygros” Hospital, School of Medicine, National and Kapodistrian University of Athens, Athens, Greece; 2 Endocrine Unit and Diabetes Centre, Department of Clinical Therapeutics, School of Medicine, Alexandra Hospital, National and Kapodistrian University of Athens, Athens, Greece

**Keywords:** Skin, Type 2 diabetes, GLP-1RA, Psoriasis

Glucagon-like peptide 1 receptor agonists (GLP-1RAs) are medications commonly used for patients with type 2 diabetes mellitus (T2DM) and obesity. Recent studies have indicated that GLP-1RAs may also benefit patients with inflammatory skin conditions, such as psoriasis. Psoriasis is a chronic inflammatory skin condition affecting millions of people worldwide. There are different types of psoriasis, but the most prevalent type is psoriasis vulgaris which is characterized by well-defined erythematous plaques covered with scales [1]. The pathogenesis of psoriasis involves the migration and activation of several immune cells, including T helper 17 cells and γδ T cells, and the secretion of proinflammatory cytokines, such as TNF-a, IL-12, IL-17, and IL-23. This inflammatory environment leads to acanthosis, parakeratosis, and dermal inflammatory infiltrates [2].

GLP-1 is an incretin hormone produced by L cells located in the ileum. GLP-1 and analogues are able to reduce blood glucose levels after a meal by stimulating insulin secretion [3]. GLP-1RAs can also act as weight-loss medications as they can delay gastric emptying and reduce appetite [4]. These effects exerted by GLP-1RAs can also be beneficial in patients with psoriasis given that T2DM and psoriasis share some pathogenic mechanisms [5]. T2DM causes chronic hyperglycemia, which is an inflammatory state and therefore can worsen psoriasis. However, there has been some evidence suggesting that the positive therapeutic effect of GLP-1RAs in psoriasis is not related to lower blood glucose levels, since other glucose-regulating medications cannot improve psoriasis [6]. Furthermore, recent studies suggest that the improvement in patients with psoriasis and T2DM treated with GLP-1RAs is present before glucose regulation or reduction in body weight [7–10]. This indicates that GLP-1Ras may act through different mechanisms.

GLP-1RAs have also been used as a treatment for obesity. Obesity is characterized by low-grade inflammation, which can worsen psoriasis [6]. Consequently, treatment with GLP-1RAs can reduce the inflammatory state and possibly improve psoriasis. The effect of GLP-1 is also evident in patients who have undergone bariatric surgery, and, specifically, Roux-en-Y gastric bypass (RYGB). Immediately after gastric bypass, GLP-1 levels are elevated and patients exhibit significant improvement in the severity of their psoriasis [11].

The positive effect of GLP-1RAs in patients with T2DM and psoriasis has been supported in several studies and can be explained by the immunoregulative and anti-inflammatory effects of these medications [7–10]. In a study by Hogan et al. [7], it was found that GLP-1RAs can affect invariant natural killer T cells (iNKT cells), which are involved in the pathogenesis of psoriasis. iNKT cells, which compose a category of natural killer T cells (NKT cells), are T lymphocytes that express both NK cell surface markers and T cell receptors (TCRs). *i*NKT cells are able to recognize lipid antigens, such as a-Gal-Cer lipid antigen, when they are presented by Cd-1d, a non-classical MHC molecule. When the TCR of the iNKT cell recognizes a lipid antigen, presented by the Cd-1d of an antigen-presenting cell, iNKT cells are stimulated. This leads to rapid production and secretion of inflammatory cytokines, chemokines, and transcription factors, such as IFN-γ, IL-2, IL-4, IL-10, IL-17, TNF-a, and GM-CSF [12]. According to Hogan et al. [7], iNKT cells infiltrate the psoriatic plaques and, as a result, circulating iNKT cell levels are lower in patients with psoriasis than in healthy individuals. A possible explanation for this phenomenon is the increased expression of Cd-1d in human keratinocytes.

In the same study [7], it was shown that GLP-1RAs can improve psoriasis severity by redistributing iNKT cells from the psoriatic plaques to the blood circulation. In a study by Faurschou et al. [8], it was found that GLP-1R may be expressed on immune cells, including iNKT cells. GLP-1R is coupled with a G-protein and, upon being stimulated, it activates adenylic cyclase and protein kinase A (PKA) by upregulating the production of cAMP [7]. This leads to phosphorylation of the transcription factor CREB, which eventually inhibits NF-kB and the production of inflammatory cytokines [7]. At the same time, phosphorylation of the transcription factor CREB increases the production of anti-inflammatory cytokine IL-10 [7]. This indicates that GLP-1RAs can target iNKT cells by reducing the production of proinflammatory cytokines and possibly increasing the production of anti-inflammatory cytokines, leading to remission of psoriasis.

Dermal γδ T cells are another category of T cells in the dermis that are involved in the pathogenesis of psoriasis. According to a study by Buysschaert et al. [13], patients with psoriasis who received GLP-1RA had a significant reduction of γδ T cells on skin biopsies after 20 weeks of treatment (from 5.9 ± 4.6% to 2.9 ± 3.5%). Dermal γδ T cells are able to produce the proinflammatory cytokine IL-17. Patients with psoriasis present with an increased production of IL-23, which can stimulate γδ T cells and the secretion of IL-17 [14].

Beyond iNKT cells and γδ T cells, GLP-1RAs have other immunological effects as well. There is some evidence that GLP-1RAs inhibit the signaling pathway of TNF-a. More specifically, GLP-1RAs inhibit NF-kB and thus decrease the production of proinflammatory cytokines, which would normally have been produced after the activation of the TNF-a receptor [9, 15, 16]. GLP-1RAs are also able to reduce lymphocyte migration. This is possibly due to the fact that GLP-1RAs can decrease the stromal cell-derived factor 1(SDF-1), which is responsible for CD4 + lymphocyte migration [17, 18]. Finally, GLP-1RAs can reduce the migration of immune cells by inhibiting the phosphatidylinositol-4,5-bisphosphate 3-kinase pathway. This leads to inhibition of signaling pathways involved in the proliferation and migration of the immune cells [15, 17, 18].

A number of researchers, having established the therapeutic benefits of GLP-1RAs in patients suffering from T2DM and psoriasis, have presented their results in several papers. As reported by Hogan et al. [7], improvement in a patient with psoriasis was observed within 48 h of treatment with exenatide. In two other patients treated with liraglutide, except for a reduction in psoriasis severity, it was found that iNKT cells were elevated in the bloodstream and decreased in psoriasis plaques. In a study by Buysschaert et al. [9], a patient with a BMI of 25.5 kg/m^2^, and HbA1c of 8.1% received exenatide and, after 12 months, his body weight, HbA1c, and severity levels of psoriasis were reduced. However, the treatment with exenatide was discontinued and, after 6 months, his psoriasis deteriorated. The patient resumed the GLP-1RAs treatment, leading to an improvement in the severity of his psoriasis as well as in glucose regulation and body weight management. In a randomized controlled trial conducted by Lin et al. [19], the control group was administered conventional treatment for psoriasis and T2DM, while the treatment group was administered liraglutide. At the end of the study, the treatment group had significantly reduced psoriasis severity and DLQI (the Dermatology Life Quality Index), and lower levels of IL-23 and IL-17, while TNF-a did not differ between groups. The positive outcome for patients with psoriasis who were treated with GLP-1RAs has been limited only in patients who also suffer from T2DM. In a randomized placebo-controlled trial by Faurschou et al. [20], liraglutide was shown to have no impact on obese patients with psoriasis who did not suffer from T2DM. In this study, GLP-1RAs acted as weight-loss medications and reduced cholesterol levels but had no effect on psoriasis severity.

To sum up, increasing data suggest that, due to their immunological effects, GLP-1RAs are valuable therapeutic options for patients suffering from T2DM and psoriasis. Further research is required to elucidate the mechanisms by which GLP-1RAs affect the immune cells and the possible impact of these medications on other inflammatory skin conditions.
